# Basic Life Support: A Questionnaire Survey to Assess Proficiency of Radiologists and Radiology Residents in Managing Adult Life Support in Cardiopulmonary Arrest and Acute Anaphylactic Reaction

**DOI:** 10.1155/2014/356967

**Published:** 2014-02-12

**Authors:** Tariq Alam, Yasir Jamil Khattak, Muhammad Anwar, Asif Alam Khan

**Affiliations:** ^1^The Aga Khan University Hospital, Pakistan; ^2^Khyber Medical College Peshawar, Pakistan

## Abstract

The aim of this paper is to assess proficiency of radiologists and radiology residents in managing adult life support in cardiopulmonary arrest and acute anaphylactic reaction.

## 1. Introduction

In the current modern era due to extensive advances in supportive care, radiology departments frequently receive critically ill patients for different procedures.

Despite supportive care for stabilization before radiologic procedures, such patients still have chances of cardiopulmonary arrests [1]. Cardiopulmonary resuscitation (CPR) in such patients is an important link in the Chain of Survival and can get life-saving time between early access to emergency care and early defibrillation.

Quick initiation of CPR, as well as high quality CPR, is crucial to survival.

Delay in commencing effective life support can result in a very poor outcome for the individual, with the highest chance of survival being, if CPR is commenced by well trained staff within 2 minutes of an arrest [2].

Though being a real important issue, there is still too less attention within the curriculum of training programs of both radiologists and radiology residents for teaching basic life support skills in an attractive way. Therefore they are not confident enough in such situations to initiate a CPR as they have not received enough satisfactory training relevant to applying CPR.

In order to assess this serious issue we conducted a questionnaire survey in Pakistan to assess ability of radiologists and radiology residents in managing adult life support in cardiopulmonary arrest and acute anaphylaxis reaction.

## 2. Material and Methods

This was a multicentre questionnaire survey used to assess knowledge of radiologists and radiology residents in managing adult cardiorespiratory arrest.

List of radiology residents was taken from the College of Physician and Surgeon, Pakistan.

124 radiologists and residents from 6 postgraduate teaching hospitals in 4 major cities of Pakistan participated and were included in the study.

A pilot study at our department was conducted to assess recent training and knowledge of adult basic life support. A questionnaire was designed for assessment of clarity and completeness in the participants. The final questionnaire was finalized without any change.

The questionnaires along with a covering letter explaining the purpose of study were sent to selected radiology residents by postal service in the respective.

The questionnaire included demographic details and following questions are related to recent training and knowledge regarding adult basic life support:

1. the most appropriate rate of CPR for an adult (chest compressions: breaths);
2. check for pulse timing;
3. maneuver that can be used to open the air way;
4. the next appropriate step, if no pulse is present;
5. the next appropriate step, if pulse is present;
6. knowledge of signs/symptoms of an adverse reaction to contrast;
7. confidence in initiating BLS;
8. assistance by staff members (radiographers, assistants, etc.) in BLS.

The radiology residents were asked to fill the questionnaire from the residents at the same time during morning or evening sessions.

The questions regarding recent training and knowledge of adult basic life support have multiple responses. The participants were asked to encircle the appropriate response.

The respondents were given an explanation that their involvement was voluntary and confidentiality would be maintained.

## 3. Results

**Figure 1:**
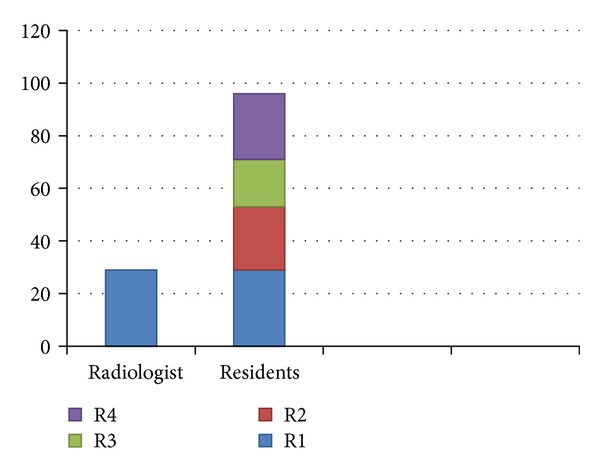


Of the 124 participants 29 were consultants and 95 were residents from year 1 to 4 (Figure 1). Nearly 44.85 percent of them answered correctly regarding basic life support. The correct responses for question one to eight were 29.0, 87.9, 74.2, 58.1, 19.4, 52.4, 20.2, and 17.7 percent, respectively. Only 29 percent of the respondents correctly told the most appropriate rate of CPR for an adult (chest compressions: breaths). Over 88 percent knew that pulse should be checked for no more than 10 seconds. About two-thirds (74 percent) told the correct maneuver that can be used to open the air way.

The majority (58 percent) knew the next appropriate step to be taken if no pulse is present.

The response for the next appropriate step if pulse is present was very poor (19.4 percent).

More than half knew the signs/symptoms of an adverse reaction to contrast.

The alarming point was that only 20.2 percent were confident to initiate BLS.

**Figure 2:**
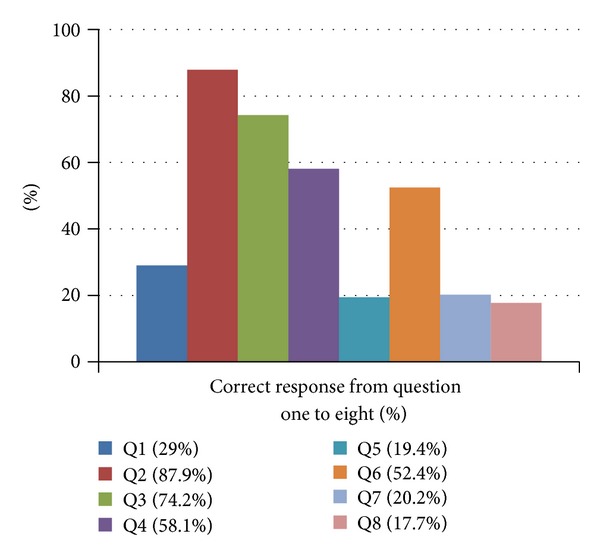


More astonishing was that only 17.7 percent felt that staff members around them (radiographers, assistants, etc.) would be able to assist in BLS (Figure 2).

Overall average score was calculated as 3.41 and 3.64 for consultants and residents, respectively.

**Figure 3:**
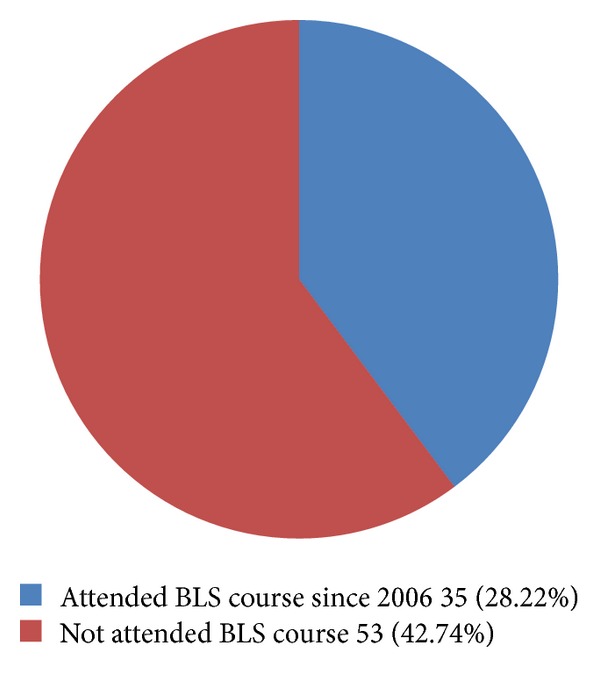


Only 35 (28.22%) responders had attended life support course since 2006 whereas 53 (42.74%) responders did not attend the course at all (Figure 3). Of them 15.1% were radiologists and 84.9% residents. Those who attended life support course more recently were more likely to respond correctly.

## 4. Discussion

Despite the fact that CPR is an important link in the Chain of Survival and can buy life-saving time between early access to emergency care and early defibrillation, our study shows that most of the radiology residents and radiologists lack knowledge of the basics of adult basic life support.

Due to extensive advances, the radiology department receives critically ill patients for different procedures. The increase in number of patients cardiac arrest at radiology department is likely to increase. Therefore it needs of time to enhance capabilities of the radiology radiologists to perform effective CPR.

Our study showed that only 35 (28.2%) responders had attended basic life support course since 2006. While 53 (42.7%) responders have not attended course at all, of them 15.1% were radiologists and 84.9% residents. Health care professionals should take notice of this alarming factor.

The main reason for the inability and underconfidence is to initiate BLS by the radiology residents and radiologist is inadequate training. It is obvious that who has attended the BLS course performed well in comparison to those who did not attend the course.

Similarly, those who attended the BLS course recently performed well in comparison to those who attended the course quite some time back. In this regards our results are comparable to Tapping and Culverwell [3].

A great percentage of the respondents did not answer correctly to the question on the last guidelines of the American Heart Association Basic Life Support, 2010 that were relevant to the appropriate ratio of thoracic compressions to ventilations.

A significant factor which reinforces radiology residents and radiologists attitude to initiate CPR and perform correctly is the working experience and attitude. Most of the time they do not come across cardiopulmonary arrest situation which requires CPR; therefore, they feel less confident to initiate or perform it. As evident from the alarming results of our study there is still too less attention within the curriculum at medical universities for teaching life support skills in an attractive way.

A better way to change this attitude and increase the confidence of radiology residents and radiologist in initiating and performing CPR is systematic CPR training programs at regular intervals and incorporating them in curriculum.

Retention of knowledge and skills during and after training in CPR is difficult and requires a more systematic training as well as methods that will ensure better retention of skills and information [4].

In a study conducted by Oh and Han 47 professional nurses were tested on their skill of CPR 3 times at an interval of 4 months. From the results, it was confirmed that re-education clearly affects nurse's knowledge and skills by maintaining their knowledge and enhancing their skills [5].

Repeating training helps staff retain knowledge in CPR [6, 7].

## 5. Conclusion

Radiology consultants and residents in Pakistan do not meet objectives of basic life support and are unable to manage adult cardio respiratory arrest scenario. BLS training programs and refresher courses should be mandatory during residency in order to acquire required competency level for BLS.

## Appendix

### Basic Life Support: A Questionnaire Survey of Radiologists and Radiology Residents in Pakistan

*Questionnaire*

Please encircle one response.

Level: consultant [].

Resident: R1, R2, R3, R4.

Last basic life support course attended: 2001–2003, 2004–2006, 2007-2008, 2009–2011.

1. The most appropriate rate of CPR for an adult (chest compressions : breaths):
  - 5 : 2, (B) 10 : 2, (C) 15 : 2, (D) 30 : 2.
2. Check for pulse for no more than
  - 10 seconds, (B) 5 seconds, (C) 15 seconds.
3. Which maneuver can be used to open the air way?
  - sweep finger in mouth, (B) head tilt-chin lift, (C) chin tilt-head lift.
4. If no pulse is present, the next appropriate step is to
  - begin chest compressions, (B) ask for help, (C) administer 2 breaths.

(5) If pulse is present, the next appropriate step is to
  - administer rescue breaths, (B) begin compressions, (C) no intervention required.
(6) Do you know the signs/symptoms of an adverse reaction to contrast?
  - yes, (B) no.
(7) Would you feel confident initiating BLS?
  - yes, (B) no, (C) do not know.
(8) Do you feel that staff members around you (radiographers, assistants, etc.) would be able to assist you in BLS?
  - yes, (B) no, (C) do not know.

## References

[1] Hope WW, Von Der Embse K, Mostafa G (2011). Cardiopulmonary arrest occurring in the radiology department: patient characteristics, incidence, and outcomes. *American Surgeon*.

[2] Zheng Z-J, Croft JB, Giles WH, Mensah GA (2001). Sudden cardiac death in the United States, 1989 to 1998. *Circulation*.

[3] Tapping CR, Culverwell AD (2009). Are radiologists able to manage serious anaphylactic reactions and cardiopulmonary arrest?. *British Journal of Radiology*.

[4] Hamilton R (2005). Nurses’ knowledge and skill retention following cardiopulmonary resuscitation training: a review of the literature. *Journal of Advanced Nursing*.

[5] Oh S-I, Han S-S (2008). A study on the sustainable effects of reeducation on cardiopulmonary resuscitation on nurses’ knowledge and skills. *Journal of Korean Academy of Nursing*.

[6] Berden HJJM, Willems FF, Hendrick JMA, Pijls NHJ, Knape JTA (1993). How frequently should basic cardiopulmonary resuscitation training be repeated to maintain adequate skills?. *British Medical Journal*.

[7] Kuhnigk H, Sefrin P, Paulus T (1994). Skills and self-assessment in cardio-pulmonary resuscitation of the hospital nursing staff. *European Journal of Emergency Medicine*.

